# Fatigue Behavior and Fracture Surface Analysis of Corroded High-Strength Bridge Cable Wires

**DOI:** 10.3390/ma17081724

**Published:** 2024-04-09

**Authors:** Zhongxiang Liu, Tong Guo, Wenjie Li, Qinghua Zhang, Bin Cheng, José Correia

**Affiliations:** 1 School of Transportation, Southeast University, Nanjing 210096, China; zhongxiang@seu.edu.cn; 2 Key Laboratory of Concrete and Prestressed Concrete Structures, Ministry of Education, Southeast University, Nanjing 210096, China; 3 Academician Office, CCCC Highway Bridges National Engineering Research Centre Co., Ltd., Beijing 100088, China; liwenjie@hpdi.com.cn; 4 Department of Bridge Engineering, Southwest Jiaotong University, Chengdu 610031, China; swjtuzqh@126.com; 5 State Key Laboratory of Ocean Engineering, Shanghai Jiao Tong University, Shanghai 200240, China; cheng_bin@sjtu.edu.cn; 6 CONSTRUCT, Faculty of Engineering, University of Porto, 4200-465 Porto, Portugal; jacorreia@fe.up.pt

**Keywords:** bridge cable wire, corrosion fatigue, fracture surface analysis, multiple pits, zinc–aluminum alloy coating, fatigue life

## Abstract

Bridge cable wires suffer from alternating stress and environmental erosion, leading to premature failure prior to its design life. This paper investigates the fatigue and mechanical behaviors of corroded bridge cable wires with a zinc–aluminum (Zn-Al) alloy coating. Based on the salt spray corrosion test and microstructure analysis, the anti-corrosion resistance and corrosion appearance characteristics of the Zn-Al alloy coating and galvanized coating were investigated. The Zn-Al alloy coating was superior in resistance to corrosion fatigue for the improvement in toughness and the generation of anti-corrosion Zn-Al and Fe-Zn-Al phases. Equations of the accelerated corrosion depth of the steel wires had been regressed to roughly estimate the corrosion life of the Zn-Al alloy coating, which can reach 29.1 years with a thickness of 70 μm. The fatigue and mechanical properties of the bare wires after the salt spray test were further studied based on tensile tests and fatigue tests. The fatigue properties of the bridge cable wire would decrease with the corrosion degree due to the deterioration and embrittlement of materials, where ductility characterized by the elongation rate was the most affected. Fracture surfaces of the wires were captured and analyzed based on a method for recognizing graphical contours. Insufficient fatigue life may occur in the steel wires after corrosion and increase with the degree of corrosion. The pit depth logarithmically weakened the fatigue life of steel wires for the weakening of fatigue toughness and the bearing area. The flat fracture was more common with a single fatigue source, while multiple fatigue sources led to step-like fractures for the generation of multiple dispersed crack propagation regions. Corrosion fatigue was more sensitive to the existence of fatigue sources than the reduction. Multiple initiation sources significantly reduced the fatigue life due to the cracking facilitation of the joint effect of multiple pits. The electrochemical reactions of corrosion can lead to material embrittlement and a reducing effect on the fracture toughness of the steel wires.

## 1. Introduction

Bridge cables are prone to corrosion due to damage and leaks in the protective layer during construction and operation. Subsequently, direct contact between corrosive medium and high-strength steel wires follows, causing the rusting of wires [1,2]. Although corrosion can be delayed by prevention measures, it cannot be completely avoided. Over time, the degradation in the performance of bridge cable steel wires due to corrosion can lead to cable failure [3,4]. Existing engineering cases have confirmed that corrosion is a major factor for the degradation of bridge cable wires in terms of mechanical properties, applicability, and durability [5,6]. Moreover, bridge cables suffer from fatigue action continuously because of the large amount of stress amplitudes caused by vehicles and wind [7]. Cracks may initiate and propagate in the steel wires under the accumulative effect of stress amplitudes, causing the fracture of steel wires. Hence, the combined action of corrosion and fatigue was involved in some cable failures of existing bridges. As the critical load-carrying parts on large-span bridges, the replacement of cables, while technically feasible, often incurs substantial costs [8,9]. Therefore, the assessment and determination of load-bearing capacity and fatigue behavior of corroded wires used in bridges are of significant value for bridge maintenance and management.

Studies related to the fatigue and corrosion properties of steel wires have been ongoing [10,11,12]. P. Roffey [13] investigated the corroded steel wires of the Forth Road Bridge based on a series of tests. The results of the metallography examination and fatigue test indicate that the reduction in tensile strength was due to localized corrosion pits. Accelerated corrosion tensile tests using a self-made stress corrosion device and finite element simulation on steel wires were conducted [14]. The degradation of the ultimate elongation of the corroded cable was confirmed to be the most significant, followed by the yield strength and the ultimate strength. Betti et al. [15] completed accelerated corrosion tests and durability analysis on the tensile steel wires of suspension bridges to study the deterioration mechanism. Moreover, some long-term exposure corrosion experiments of bridge cables were executed to figure out the impact of wet and dry environmental conditions [16]. The rusted high-strength wires were classified into four categories according to the environment, and high temperature and moisture in the cables were considered to be the main causes of rusting. Li et al. [17] performed artificially accelerated rusting on bridge steel wires to evaluate the development of uniform rust and pit with time in depth, and they established a statistical distribution model of the pit corrosion coefficient. Lan et al. [18] proposed a model to express the evolution rule of fatigue damage for bridge steel wires by acid salt spray corrosion and fatigue loading tests, showing that more severe corrosion resulted in shorter fatigue life. The fatigue mechanisms and life degradation of bridge cable wires were investigated by Guo et al. [19], revealing that the geometric irregularity and discontinuity in the wire surface provided a cracking source and stress concentration. In addition, life prediction models for steel wires considering the effect of fatigue or corrosion were developed [20]. The above studies provide a preliminary basis for understanding the degradation of fatigue behavior of high-strength galvanized steel wires and establishing the life evaluation method [21,22,23]. However, investigation on the fatigue behavior of zinc–aluminum (Zn-Al) alloy-coated steel wires under corrosion action is insufficient. Furthermore, the fatigue behavior analysis of corroded steel wires is deficient in the aspect of fracture morphology.

The present work investigates the fatigue behavior of corroded high-strength steel wires with a Zn-Al alloy coating according to experiments and fracture surface analysis. Acetic acid salt spray corrosion tests are executed to analyze the corrosion behavior of bridge cable wires with galvanization and a Zn-Al alloy coating. The anti-corrosion resistance and corrosion appearance characteristics of the Zn-Al alloy coating and galvanized coating are investigated based on the salt spray corrosion test and microstructure analysis. The fatigue behavior and mechanical properties of bridge cable wires subjected to different corrosion degrees will be investigated via mechanical and fatigue experiments. Fracture surfaces are further analyzed by using a high-precision industrial camera and a method for recognizing graphical contours. The geometrical dimensions and morphologies of fatigue fracture of the rusted wires are extracted and analyzed and the influence law of the corrosion degree on the fatigue behavior of corroded wires used in bridges will be further discussed.

## 2. Materials and Methods

### 2.1. Materials

**Figure 1:**
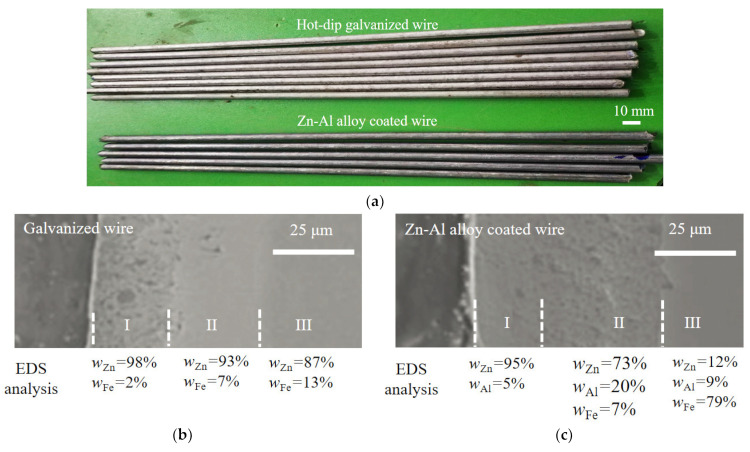
Specimens of steel wires and element content analysis of two coatings: (**a**) Specimens of steel wires; (**b**) microstructure and element content analysis of galvanized coating; (**c**) microstructure and element content analysis of Zn-Al alloy coating.

Bridge cable wires with galvanization and a Zn-Al alloy coating were used here (manufacturer: Jiangsu Fasten Co., Ltd., Jiangyin, China). The Zn-Al alloy coating is an alternative to the traditional pure zinc coating due to its good corrosion resistance [24,25]. To investigate the fatigue behavior of bridge cable wires, steel wires with a hot-dip galvanized coating and Zn-Al alloy coating were selected, with diameters of about 5.0 mm and 5.4 mm, respectively. The two types of specimens are shown in Figure 1a, whose tensile strengths are no less than 1670 MPa. The specimens were all 300 mm in length. The diameter of the specimens with a hot-dip galvanized coating and Zn-Al alloy coating were 5.0 mm and 5.4 mm, respectively. The chemical composition of the bare steel wires of all specimens studied is listed in Table 1.

The microstructure and element composition of the hot-dip galvanized coating and Zn-Al alloy coating of the specimens were further studied by a scanning electron microscope (SEM) and energy density spectrum (EDS). Figure 1b shows the SEM picture and EDS result, where *W*_Zn_, *W*_Fe_, and *W*_Al_ present the weight percent of the elements zinc (Zn), iron (Fe), and aluminum (Al). The thicknesses of the zinc coating and Zn-Al alloy coating were about 50 μm and 70 μm, respectively. According to Figure 1b,c, the two types of coatings can both be divided into an outer layer, intermediate layer, and inner layer, numbered I, II, and III. For the galvanized coating in Figure 1b, the mass fraction of Zn in layer I was about 98%, which can be determined as a pure Zn phase. The mass fraction of Zn in layer I was about 98%, which can be determined as a pure Zn phase. The molecular ratio of Zn to Fe in layer II was about 12, which is close to the molecular ratio of FeZn_13_. Similarly, the molecular ratio of Zn to Fe in layer III was about 6, which is close to the molecular ratio of FeZn_7_. For the Zn-Al alloy coating in Figure 1c, the mass fraction of Zn in layer I was 95% slightly lower than that in the layer I of the galvanized coating. Moreover, the mass fraction of Al in layer I of the Zn-Al alloy coating was 5% without the Fe element. As the molecular ratio of Zn and Al in layer I was about 8:1, this layer was mainly composed of Zn-Al eutectic and alloy phases. The mass fraction of Zn, Al, and Fe in layer II of the Zn-Al alloy coating revealed the existence of the Zn-Al-Fe alloy phase. In the comparison of the two coatings in Figure 1b,c, the microstructure of the Zn-Al alloy coating was denser than that of the pure zinc coating. This was probably because the element Al can effectively inhibit the formation of relatively loose Fe-Zn and pure Zn phases and generate dense and uniform Zn-Al and Fe-Zn-Al phases with better corrosion resistance. Moreover, the addition of the element Al can significantly reduce the thickness of the intermediate layer, which can greatly improve the toughness of the coating.

### 2.2. Salt Spray Test of Steel Wires

**Figure 2:**
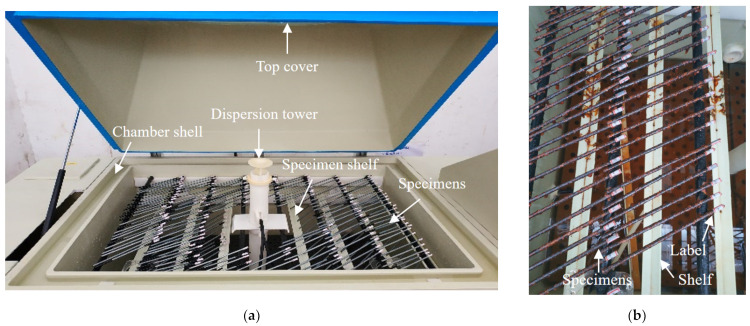
Salt spray test and specimens: (**a**) test arrangement; (**b**) corroded specimen.

Neutral and acid salt spray tests are commonly utilized to evaluate the corrosion resistance of materials, coatings, or specimens [26,27,28], where the acid salt spray test is more rigorous for acidic environments [29]. The addition of acetic acid can accelerate the corrosion process, with an acceleration effect three times faster than that of a neutral environment [30]. To obtain corroded steel wires and investigate the corrosion resistance of the coatings, salt spray tests were conducted in this study. The arrangement of the salt spray tests is shown in Figure 2a, where a salt spray test chamber was used (mode: YWX/Q-250, manufacturer: Nanjing Test Equipment Co., Ltd., Nanjing, China). The operating conditions were designed as follows: (1) the spray pressure was controlled at about 0.1 MPa; (2) corrosion solution was at pH 3.1 to pH 3.3; (3) the test was performed 24 h per day without interruption; (4) the test ambient temperature was set to 35 °C; (5) an average sedimentation rate of the horizontal surface area was set at 1.5 mL/h ± 0.5 mL/h. During the salt spray tests, the specimen was flipped and repositioned once every 100 h (approximately every 4 days) to ensure similar levels of corrosion on both sides of the specimen. The corroded specimens in the salt spray chamber are presented in Figure 2b. The specimens were labeled and cling film was wrapped around the ends of the specimens to prevent the corrosion of the labels.

To maintain the pH value, a 5% solution of acetic acid was used as the test solution and the test chamber was maintained at a certain level of acidity. According to GB/T 10125-2021 [31], the water conductivity of the corrosion solution was required not to exceeded 20 μS/cm, which can be prepared by sodium chloride and distilled water. The concentration was prepared at 50 g/L ± 5 g/L, and the solution density was approximately 1.05 g/cm^3^ at a preparation temperature of 25 °C. Then, the corrosion solution was adjusted to pH 3.1 by adding an amount of glacial acetic acid. According to GB/T 17101-2019 [32], the pickling solution was prepared as a cleaning solution for removing corrosion products. To prevent the corrosion liquid of the upper layer from dripping onto the lower layer, all specimens were placed horizontally on the upper or lower shelf of the salt spray chamber in a staggered manner. The specimens were horizontally arranged at approximately 25° with the salt spray chamber. The arrangement schematic of the specimens is shown in Figure 2a.

The specimens were naturally dried under indoor conditions for 0.5–1 h and then dried using a cold air blower. A plastic brush was used to clean the surface corrosion residues. For specimens that did not enter the corrosion stage of bare steel wire, the surface corrosion products were cleared using sandpaper with a grit size of 1000 or higher. Then, the surface was rinsed with clean water and dried with a cold air blower. This process was repeated several times until there were no white impurities remaining on the surface. For specimens that entered the stage of bare steel wire corrosion, the surface was first rinsed with clean water. Then, it was immersed in a properly prepared pickling solution for 10 min. After removal, it was thoroughly rinsed with clean water and immediately dried using a cold air blower.

### 2.3. Tensile and Fatigue Tests of Steel Wires

**Figure 3:**
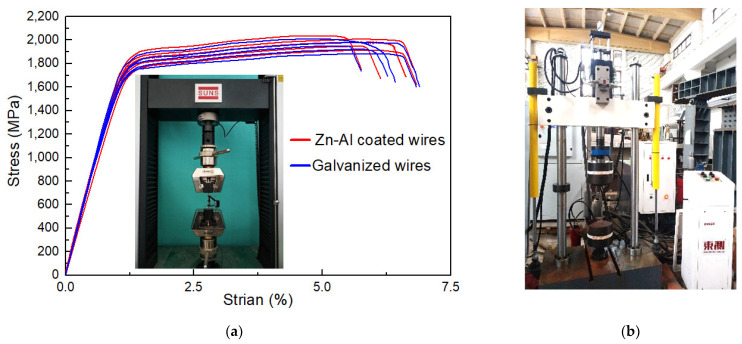
Tensile and fatigue testing of specimens with different mass loss rates: (**a**) static tensile test and stress–strain curves; (**b**) fatigue test.

To investigate the influence of corrosion on the mechanical properties, static tensile tests were conducted on the specimens, as shown in Figure 3a. A 5-ton testing machine (mode: UTM5000, manufacturer: Shenzhen Senstest Equipment Co., Ltd., Shenzhen, China) was utilized. The tensile rate was controlled according to force. An extensometer (mode: Epsilon 3542, manufacturer: Epsilon Technology Company, Jackson, WY, USA) was utilized to measure stress–strain data, whose measuring length was 50 mm. Five un-corroded specimens (i.e., specimens with mass loss rate of 0%) of the steel wire with Zn-Al alloy coating were tested to obtain the initial mechanical properties. For comparison, another five un-corroded specimens of the steel wire with galvanized coating were also tested. Figure 3a illustrates the stress–strain curves of the two types of the bare steel wires, i.e., mass loss rates equal to 0%. The main mechanical properties of steel wires with the galvanized coating and Zn-Al alloy coating are shown in Table 2, including the average value and corresponding confidence interval of tensile strength (*σ_u_*), yield strength (*σ_y_*), elongation rate (*δ*), and elastic modulus (*E*). The elastic modulus can be determined according to the data from the endpoints and midpoint of the linear segment of a stress–strain curve.

Moreover, fifty-five specimens were stretched after the salt spray test to investigate the influence of corrosion on their mechanical properties. As the Zn-Al coating and surface layer of the specimens had been corroded via the salt spray test, the diameter of those specimens was 5.26 mm. The mass loss rate for those corroded specimens ranged from 0% to 21.38%, where the mass of the coating was not considered. Moreover, five other un-corroded specimens (i.e., specimens with coating and mass loss rate of 0%) were tested as controls.

To further investigate the influence of corrosion on the fatigue properties, fatigue tests were implemented on corroded specimens. A fatigue testing machine (mode: PWS-100, manufacturer: Jinan Docer Technology Co., Ltd., Jinan, China) was utilized, as presented in Figure 3b. The maximum dynamic loading capacity of the testing machine was 8 tons. Force control was selected as the test loading type, and the sine wave loading was adopted. The loading frequency was set at 10 Hz. The specimens with a mass loss rate ranging from 0% to 23% were used for the fatigue test. The fatigue life and fracture toughness were calculated. Moreover, fracture surface analysis was conducted. The test load values were determined based on GB/T 17101-2019 that the steel wire should be able to withstand 2 million fatigue cycles of the load ranging from 0.45*F_m_* to (0.45*F_m_* − 2Δ*F_a_*) without fracturing [32]. Here, we use the following:

(1) $$\Delta \sigma =\frac{2\Delta F_{a}}{S_{n}}$$

where *F_a_* represents the tensile strength of the wire, with an average measured value of 42.03 kN; *S_n_* is the cross-sectional area, with a value of 21.72 mm^2^ for the bare wire cross-section; and 2Δ*F_a_* represents the load amplitude. The calculated cyclic load combination for force control is an average force of 12.413 kN and a load value of 7.818 kN for the stress amplitude. Hence, Δ*σ* is equal to 360 MPa.

## 3. Results and Discussion

### 3.1. Corrosion Performance of Coatings and Steel Wires

#### 3.1.1. Corrosion Appearance Characteristics

**Figure 4:**
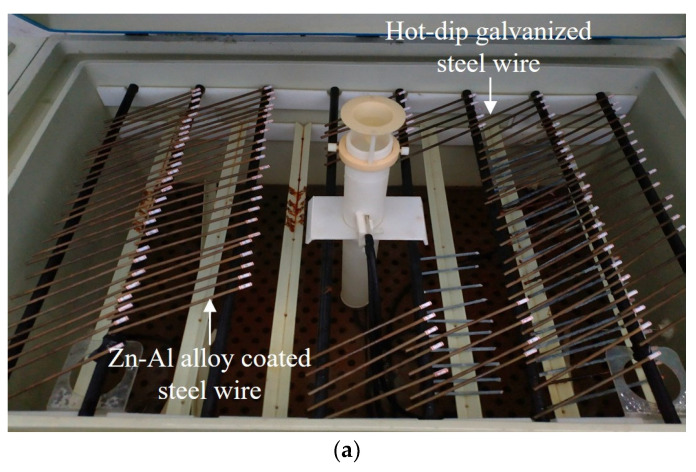

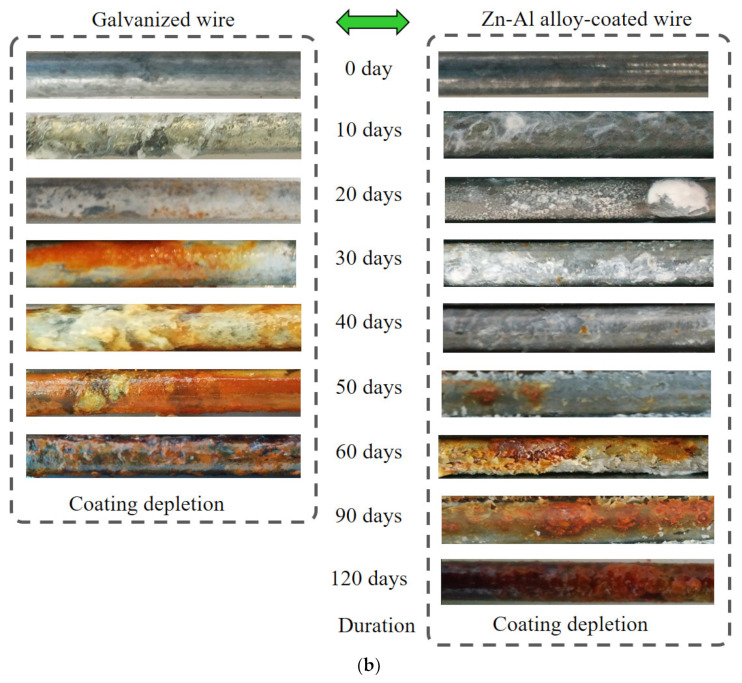
Comparison of corrosion appearance of steel wires with two different coatings: (**a**) test condition; (**b**) corrosion appearance.

The salt spray test was conducted mainly to analyze the corrosion state of the Zn-Al alloy-coated steel wires. The test conditions are shown in Figure 4a, whose details are described in Section 2.2. Comparative analysis of the salt spray resistance of the galvanized steel wires was also conducted. The surface morphologies of the corroded steel wires with two types of coatings are presented in Figure 4b, which shows the middle segment of the specimens. Considering the variation in corrosion levels among different specimens within the same corrosion period, only the wire corrosion appearance that reflects the majority of the current stage of corrosion was chosen. The zinc coating was completely depleted about 60 days after the beginning of the experiment. The Zn-Al alloy coating was completely depleted about 120 days after the beginning of the experiment. Hence, the results used for the analysis of the coating effect were measured over about 60 days and 120 days after the beginning of the experiment, respectively.

Before the start of salt spray corrosion, the galvanized steel wires appeared lighter in color compared to the steel wires with Zn-Al alloy coating. The galvanized steel wires as well as Zn-Al alloy-coated steel wires had a certain level of metallic luster without corrosion. By the 10th day, the galvanized coating lost the metallic luster, and the wire surface became rough with visible salt deposits. The Zn-Al alloy coating retained the metallic luster with minimal salt deposits. By the 20th day, yellow rust spots began to appear on the surface of the galvanized steel wire, which indicates the failure of the galvanized coating. The Zn-Al alloy-coated wire had dense white spots with salt accumulation in some areas. By the 30th day, the area of yellow rust spots on the galvanized coating continued to expand and gradually turned into a yellow-brown color. The white corrosion products accumulated further on the Zn-Al alloy-coated wire. By the 40th day, the area of rust products on the galvanized coating wire continued to expand. The Zn-Al alloy-coated wire had yellow rust spots, and there was a slight peeling of the coating surface. By the 50th day, the galvanized coating was completely covered with red rust. The area of yellow rust spots on the Zn-Al alloy-coated wire gradually increased. The iron substrate of the galvanized steel wire was exposed by the 60th day with the surface covered in black and reddish-brown iron rust. The significant presence of red rust spots and noticeable peeling of the coating occurred on the Zn-Al alloy-coated wire. The red rust formed continuous patches on the surface of the Zn-Al alloy-coated wire by the 90th day. By the 120th day, reddish-brown iron rust fully covered on the surface of the Zn-Al alloy-coated wire.

#### 3.1.2. Mass Loss Rate and Corrosion Depth of Coatings

The specimens were taken out from the test chamber and corrosion products were removed by pickling solution at the end of each exposure period. The corrosion level of the steel wires can be evaluated by the mass loss rate according to Equation (2).

(2) $$r=\frac{m_{0}L_{c}-m_{c}L_{0}}{m_{0}L_{c}}$$

where *r* is the mass loss rate; *m*_0_ is the initial mass; *m_c_* is the mass after the removal of corrosion products; *L*_0_ is the specimen’s initial length; and *L_c_* is the length of the corrosion zone.

**Figure 5:**
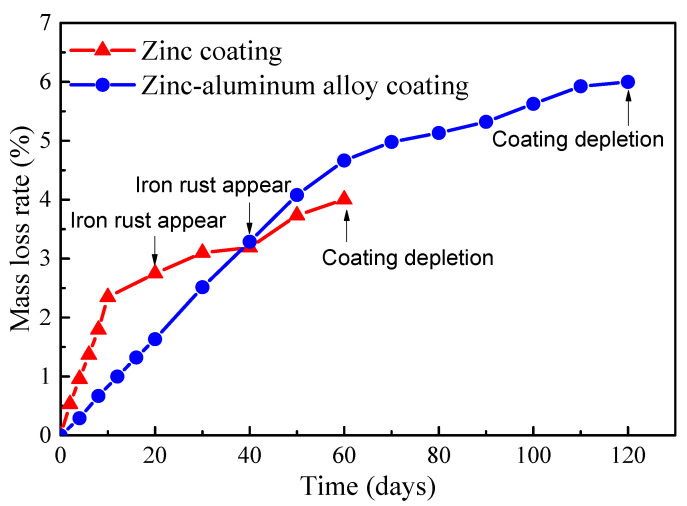
Time-varying mass loss rates of the coatings of bridge cable wires.

Quantitative analysis was conducted on the corrosion of the steel wires with a Zn-Al alloy coating over a period of 0–120 days and the galvanized steel wires over a period of 0–60 days. The time-varying mass loss rates of the two types of steel wires are illustrated in Figure 5. It can be observed that the Zn-Al alloy coating exhibited approximately twice as much salt spray corrosion resistance compared to the galvanized coating with almost the same coating thickness. Additionally, both the galvanized coating and Zn-Al alloy coating showed a rapid corrosion rate in the initial stage followed by a slower rate. It should be noted that the unevenness of the coating, caused by the coating process, may lead to the early exposure of the bare steel wire to the atmosphere and corrosion medium, resulting in the premature appearance of reddish-brown iron rust. This is the main reason for the curves in Figure 5 to exhibit a turning point. Moreover, the accumulation of rust products impeded the transfer of oxygen and metal ions in the development of subsequent corrosion, leading to a decrease in the corrosion rate in the later stages of Figure 5.

The corrosion of a steel wire with coating involves the corrosion of the coating and bare steel wire [33]. Due to the low reactivity of iron and the coverage of corrosion products, the corrosion rates in these two stages are significantly different. The average corrosion depth can be calculated by Equation (3).

(3) $$d_{ave}=\frac{m_{0}L_{c}-m_{c}L_{0}}{\rho \pi DL_{c}L_{0}}$$

where *d_ave_* is the average corrosion depth; *ρ* is the density; and *D* is the diameter of the steel wire. Here, the density of the bare steel wire is 7.85 × 10^3^ kg/m^3^, and pure zinc has a density of 7.14 × 10^3^ kg/m^3^. The density of the Zn-Al alloy coating can be calculated as 6.60 × 10^3^ kg/m^3^ according to the principle of a 5% mass fraction of aluminum and a 95% mass fraction of zinc.

**Figure 6:**
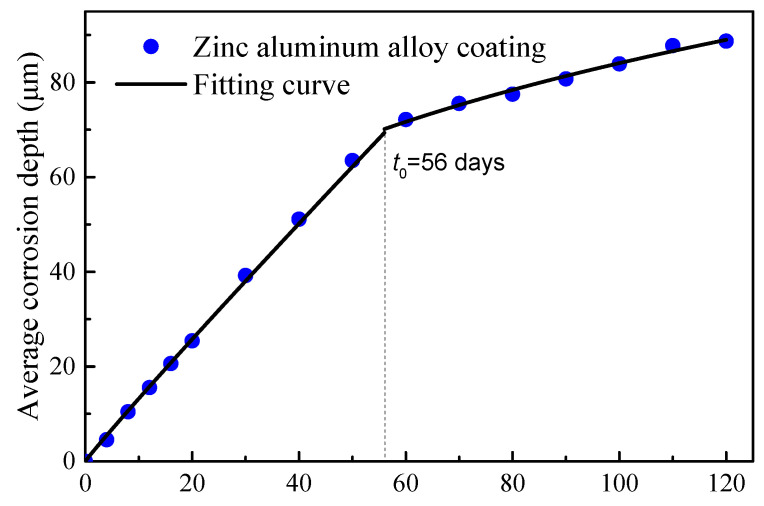
Time-varying average corrosion depth of Zn-Al alloy-coated wires.

A two-stage time-varying model of the average corrosion depth was introduced to characterize the time-varying corrosion depth. The curve for the steel wire with Zn-Al alloy coating is illustrated in Figure 6. A fitting was performed for the first stage of the Zn-Al alloy coating (data points taken up to 40 days, where the appearance of rust spots indicated the depletion of the coating for some wires). The fitted curve is given by Equation (4).

(4) $$d_{ave,Zn-Al}=1.44345t^{0.962}$$

where *d_ave_*_,Zn-Al_ is the average corrosion depth of the coating; *t* is the duration of corrosion.

It can be seen from Figure 6 that there was a turning point in the data points for the Zn-Al alloy coating in the range of 50 to 60 days, indicating the corrosion of the bare steel wire. The actual coating mass per unit area of this batch of wires was measured to be approximately 460 g/m^2^. According to the Equation (4), the calculation indicates a time of 56 days. Therefore, the mass loss of the Zn-Al alloy coating steel wire should be calculated according to the density of iron after 56 days. Compared with the turning point for the galvanized coating after 15 days of corrosion in Figure 5, the corrosion resistance life of the Zn-Al alloy layer was more than twice that of the galvanized layer with the same coating thickness. Moreover, the corrosion life of the galvanized coating with 50 μm thickness in an atmospheric environment was validated to be about 7.8 years. According to the simple equivalent conversion between the accelerated corrosion results and atmospheric corrosion results, the corrosion life of the Zn-Al alloy coating with a 70 μm thickness can reach 29.1 years.

A fitting was further performed for the corrosion stage of the bare steel wire (data points taken up from 60 to 120 days). The fitting equation for this stage is as follows:

(5) $$d_{ave,Fe}=0.58967{\left(t-t_{0}\right)}^{0.842}$$

where *d_ave_*_,Fe_ is the average corrosion depth of the bare steel wire; *t*_0_ is the corrosion duration of the coating, which is equal to 56 days according to the experiment data here.

#### 3.1.3. Mechanical Properties of Corroded Steel Wire

**Figure 7:**
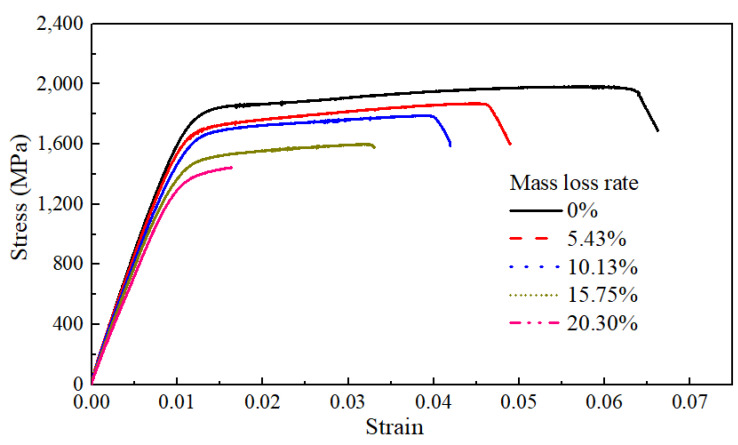
Static tensile test and analysis of bare steel wires with different mass loss rates.

Figure 7 illustrates typical stress–strain curves of the bare steel wires with different mass loss rates for corrosion, whose corrosion degrees ranged between 0% and 20.30%. It can be found that the yield platform of the steel wire shortened with a larger mass loss rate. It is indicated that some mechanical properties of the steel wire were significantly affected by the corrosion degree.

According to specification [32], the yield strength of a high-strength steel wire is set as the strength at a non-proportional elongation of 0.2% of a stress–strain curve, i.e., *R*_0.2_. Since the static tensile test was conducted by force-controlled loading, the actual strength of the rusty steel wire was calculated by determining the effective cross-section area (*S_c_*) of the wire. Hence, Equation (6) can be used for calculating the actual strength of the wires.

(6) $$S_{c}=\frac{\left(1-r\right)\pi D^{2}}{4}$$

**Figure 8:**
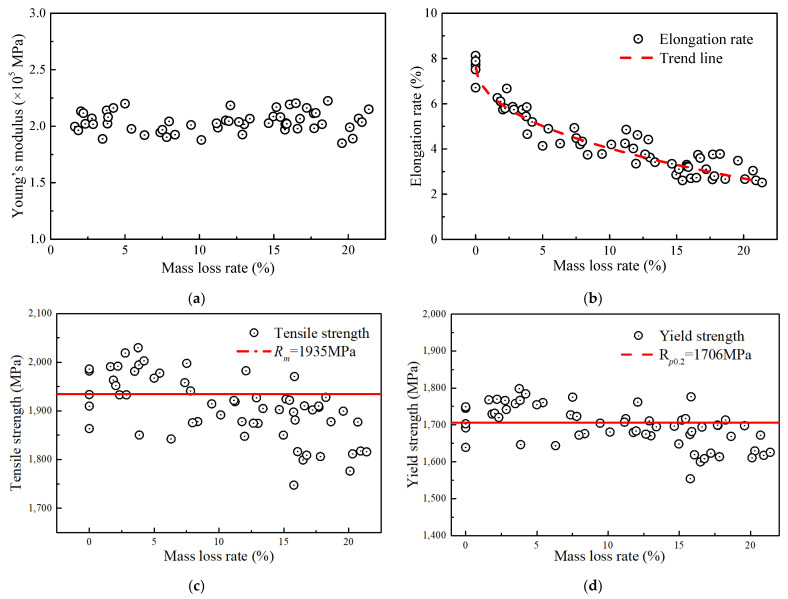
Relationship between mechanical properties and mass loss rate: (**a**) Young’s modulus; (**b**) elongation rate; (**c**) tensile strength; (**d**) yield strength.

Figure 8 shows the relationships of Young’s modulus, tensile strength, yield strength, elongation, and ultimate strain of the steel wire to the corrosion level. Figure 8a shows that the Young’s modulus of wires with different corrosion degrees fluctuated between 1.8 × 10^5^ and 2.2 × 10^5^ MPa with an average value of approximately 2.04 × 10^5^ MPa. This means that the corrosion action had a slight impact on the Young’s modulus of the wire, which is consistent with the findings reported by Liao [14]. The elongation was the most susceptible property of the steel wire to corrosion, as illustrated in Figure 8b. Within the mass loss rate range of 0% to 5%, the elongation value decreased rapidly, dropping from 7.88% to approximately 5.01%. Afterward, the change rate in elongation began to slow down. When the mass loss rate was between 5% and 20%, the elongation decreased from approximately 5.01% to about 2.70%.

According to Figure 8c,d, the actual tensile strength (i.e., *R_m_*) and yield strength calculated by using Equation (5) were affected by corrosion. Compared with the non-corroded steel wire, both the two strengths of the corroded steel wire had a gradual decrease, and those average values were measured as 1935 MPa and 1706 MPa, respectively. Before the mass loss rate reached approximately 10%, the calculated strength points based on Equation (5) were relatively stable, scattered around the average measured strength of the non-corroded wire. After the mass loss rate exceeded 10%, the strengths generally fell below the average values. For the tensile strength, the deviation was approximately within the range of 0 to 180 MPa, corresponding to a cross-sectional loss of approximately 9.30%. For yield strength, the deviation is approximately within the range of 0 to 160 MPa, corresponding to a cross-sectional loss of 9.38%. The result shows that the measured strengths were lower in the later stage due to the fracture occurring at the location of the minimum cross-sectional area. This could be the reason for the strength calculated by Equation (5) being lower than the average value.

**Figure 9:**
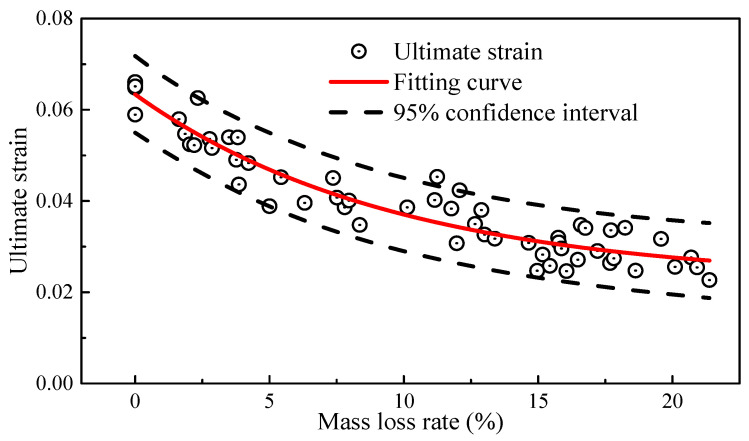
Ultimate strain and mass loss rate.

As shown in Figure 9, the ultimate strain also exhibited a negative correlation with the mass loss rate, similar to the trend observed in elongation. For the constitutive relationship model of high-strength steel wires used in bridges, the commonly used model was the bilinear four-parameter elastic-plastic constitutive relationship [34]. The constitutive equation is shown in Equation (7).

(7) $$\left\{\begin{array}{l} \sigma =E\varepsilon ,\varepsilon \leq \varepsilon_{y}\\ \sigma =\sigma_{y}+\frac{\sigma_{u}-\sigma_{y}}{\varepsilon_{u}-\varepsilon_{y}}\left(\varepsilon -\varepsilon_{u}\right),\varepsilon_{y}<\varepsilon <\varepsilon_{u}\\ \sigma =E\varepsilon ,\varepsilon \leq \varepsilon_{u} \end{array}\right.$$

where the average value of *E* = 2.04 × 10^5^ MPa; the average value of *σ_y_* = 1706 MPa; and the average value of *σ_u_* = 1935 MPa. *ε_u_* represents the ultimate strain, which is fitted to the mass loss rate using an exponential function. As shown in Figure 9, the fitting coefficient, i.e., *R*^2^, is about 0.90, and the 95% confidence interval covers most of the scattered data points, which indicates a good fit and a satisfactory fitting result. The fitted equation is as follows:

(8) $$\varepsilon_{u}=0.02242+0.04095e^{\frac{r}{0.097}}$$

In general, after being subjected to sustained fatigue loads, the damage to the specimens gradually accumulated, leading to degraded mechanical properties. This reduction in strength was further enhanced when coupled with corrosion-induced damage. However, static tensile tests on aged cable wires had shown that corrosion fatigue damage cannot affect the constitutive characteristics of the actual bridge wires [35]. The corrosion effect was manifested primarily in mass loss, with no significant changes in physical and chemical properties, including strength and elastic modulus. The affected aspect was primarily the ductility. Therefore, the following experience should be considered: (1) the obtained constitutive relationship based on corroded wires can be approximately used for the analysis of the mechanical properties of the corroded wires without considering the fatigue influence or under low cycle stress amplitudes; (2) moreover, promoting degradation due to corrosion fatigue should be considered for the analysis of cables in the bridge after long-term service.

### 3.2. Corrosion Fatigue Behavior of Steel Wires

#### 3.2.1. Corrosion Fatigue Life

**Figure 10:**
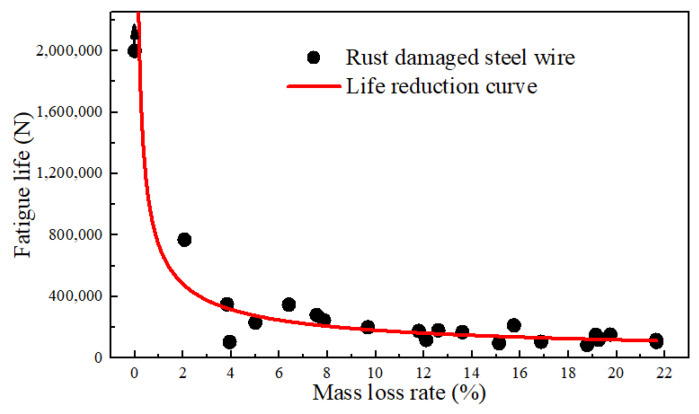
Relationship between the fatigue life and the mass loss rates.

The relationship between the fatigue life and corresponding mass loss rate of some specimens was developed, as depicted in Figure 10. The fatigue life of the un-corroded steel wire under the specified conditions was based on truncated data of 2 million cycles. The fitted life reduction curve indicates that once the steel wire was corroded, its life immediately decreased. For example, when the rust rate was 1.86%, although the surface pits were not significant, the fatigue life was already reduced to around 800,000 cycles. At a rust rate of 10%, the fatigue life approached 250,000 cycles. At a rust rate of 20%, the life reduction was around 100,000 cycles. It reveals that the interaction of fatigue stress and corrosion action can greatly reduce the life of steel wires. This impact increased with the degree of corrosion while the increase was logarithmic. It is indicated that corrosion fatigue was more sensitive to the fatigue source resulting from corrosion than the reduction in the cross-section caused by corrosion. Hence, the initial corrosion resistance and the integrity of the anti-corrosion coating of the wire were the most critical factors. Considering the requirement of a 2 million-cycle life (i.e., fatigue safety life), the corrosion of the steel wire would immediately lead to corrosion of the bare steel wire after the depletion of the galvanized layer. In a near-sea environment, chloride ions, oxygen, and suitable temperature and humidity can rapidly cause a mass loss rate of about 2% for the bare wire. Under this condition, the wire would be reduced significantly in the fatigue properties. Therefore, it is recommended to replace the wire once severe corrosion occurs.

#### 3.2.2. Fracture Surface Analysis

**Figure 11:**
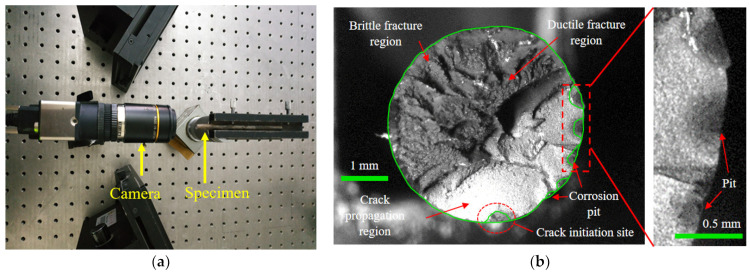
Fracture morphology testing of corroded steel wire: (**a**) arrangement of morphology testing; (**b**) step-like fracture.

After the microscopic evolution of corrosion pits into macroscopic forms, the possibility of cracking initiating from the pits gradually increases [36,37]. To investigate the corrosion fatigue fracture and the influence of the corrosion pit, fracture surface analysis was conducted. A high-precision IDS industrial camera with a 25 mm extension tube and KOWA lens was used to capture images of the fracture surfaces, as shown in Figure 11a. The fracture morphologies of the steel wires were measured. Figure 11b presents a typical one, where the crack initiation site, crack propagation region, ductile fracture region, and brittle fracture region can be clearly distinguished. There were several pits at the edge of the fracture surface, which can develop into the crack initiation sites under cyclic loading. The crack propagation region was less than half of the fracture surface, while the fracture regions accounted for over half of the fracture surface. The fracture morphology of the cross-section of the corroded steel wire can be recognized by graphical contours. According to the results of recognizing graphical contours, corrosion propagated in the circumference as well as the depth of the cross-section, meaning there was a coexistence of uniform and pitting corrosion. The corrosion progress of steel wires was dominated by pitting corrosion and multiple corrosion pits were generated, which can be evidenced by the difference in corrosion depth. This phenomenon is probably due to the unevenness of the coating and the distribution of corrosive media on the outer surface of a steel wire. The fatigue initiation sites on the fracture surfaces were mainly found at the center and vicinity of the bottom of the pit, rather than at the boundary between the pit and the steel wire surface.

**Figure 12:**
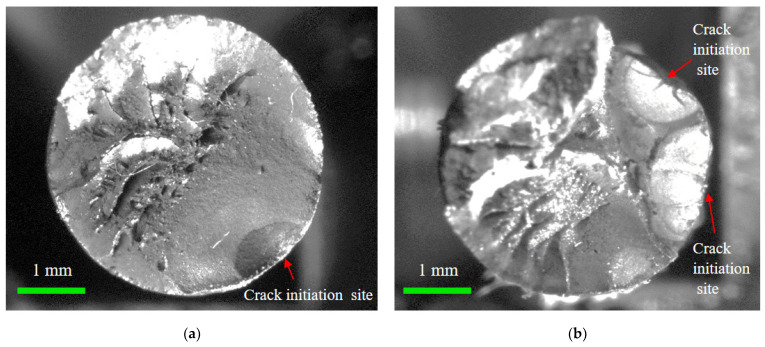
Flat and step-like fracture morphology of corroded steel wire: (**a**) flat fracture; (**b**) step-like fracture.

Moreover, the fracture morphology of the corroded steel wire can be classified into a flat type and step-like type, taking the crack propagation zone as the reference, as shown in Figure 12. The flat fracture had a small fluctuation in morphology and was more common with a single fatigue source, indicating that the cracking initiated and propagated in almost the same plane. The step-like fracture was probably caused because cracking initiated in multiple fatigue sources and propagated in multiple surfaces [38]. Hence, the reason for these different fracture morphologies was mainly due to the generation of multiple dispersed crack propagation regions from the multiple fatigue sources. For the fatigue with multiple sources, the number of fatigue sources was mostly two to three, with two being the main number. Although the area of the crack propagation region in Figure 12a approximated to that in Figure 12b, the crack propagation duration of corroded wires due to fatigue sources was greatly reduced for the simultaneous development of multiple cracking sources. As crack initiation and propagation accounted for most of the failure process of corroded wires, short fatigue life can be attributed to the weakening effect of pitting corrosion and the simultaneous development of multiple cracking sources.

**Figure 13:**
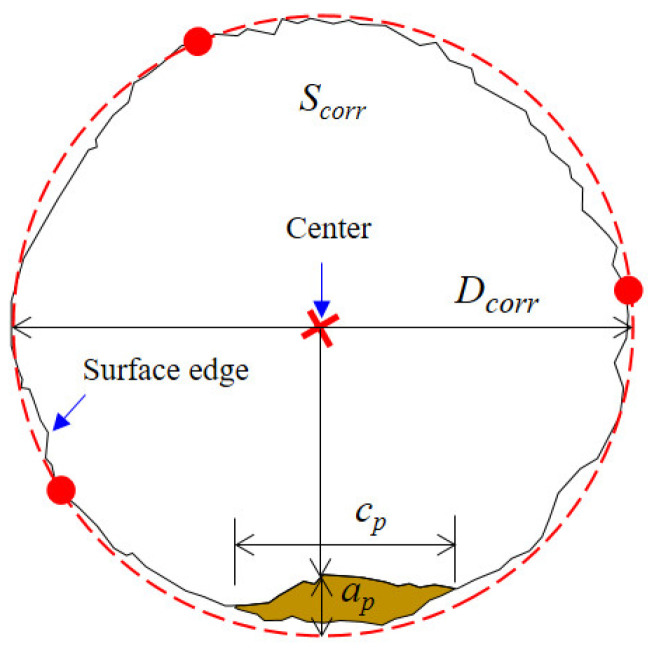
Profile regression and pit size measurement.

The two-dimensional dimensions of the pits on the fracture surface were quantitatively extracted, as shown in Figure 13. The dimensions of a pit mainly include the pit depth (*a_p_*) as well as pit width (*c_p_*). Since the steel wire surface was uneven due to corrosion dissolution, the fracture surface was not a regular circle. Therefore, it is difficult to obtain an idealized section diameter and maximum crack depth. In this study, considering that the surface was relatively smooth and represented a uniformly corroded area, three points on this surface were selected to determine a circle. This regression based on three different points was conducted multiple times. The one with the largest diameter was taken as the profile of the uniformly corroded region *S_corr_*. The diameter of this circle was denoted as *D_corr_*. Since the pits were usually small, the circle formed by the three points should be able to envelop most of the corroded surface area. The distance from the center of the circle to each point on the inner boundary of the pit was calculated. The deepest pit depth can be determined as the point with the shortest distance to the pit wall. By extending the outermost line of the fracture surface, the distance of extension represented the pit depth. The pit width was treated as the straight-line distance between the two pit ends. Pit width was linearly correlated with depth within a certain range.

Tested results indicate that the pit depth on the fracture surface followed a normal distribution N(0.254, 0.094). Since the inner wall of the pit was not a smooth ellipsoidal surface and there were no pits with identical geometric characteristics, the macroscopic pit depth can be used as the primary indicator for evaluating the effect of pits on fatigue life. Fracture life, pit profile, and the crack initiation site of the specimens were counted to evaluate the influence of pits on the failure of corroded steel wires. The statistical results indicate that the cross-sectional shape of the pit along the wire length tended to be flattened macroscopically as the mass loss rate increased. Stress concentration induced by the pit was not very pronounced, while the pit depth-to-diameter ratio of the pit was larger for specimens with a shorter life.

**Figure 14:**
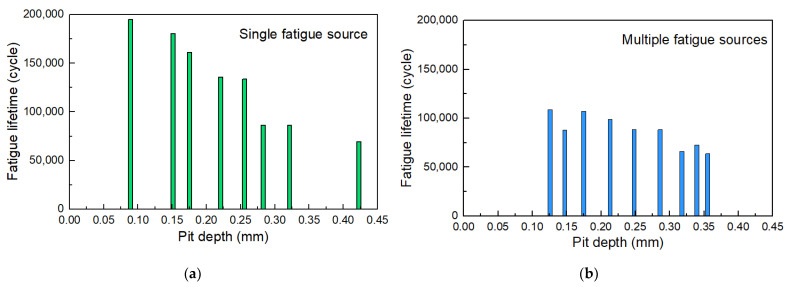
Fatigue life of corroded steel wires related to pit depth and number: (**a**) single fatigue source; (**b**) multiple fatigue sources.

Figure 14 shows the fatigue life of some specimens to figure out the influence of the pit depth on the fatigue life of corroded steel wires. It is observed that the tested fatigue life of corroded steel wires was lower than 2 × 10^6^ fatigue cycles (i.e., fatigue safety life). This shows that insufficient fatigue life may occur in the steel wires used in actual engineering after corrosion, which should be of concern. The fracture of specimens can be attributed to crack propagation from a single fatigue source and multiple fatigue sources. Figure 14a shows that the pit depth logarithmically weakened the fatigue life, where the steel wires had been corroded with different corrosion mass loss values (i.e., corrosion degree). This may be because deeper pits further weaken the fatigue toughness and bearing area. Compared with the fracture due to a single fatigue source propagation, the presence of multiple fatigue sources led to a certain reduction in life, as shown in Figure 14b. The joint effect of multiple pits facilitated crack initiation and propagation, which should be one reason. This phenomenon became less significant when the pit deepened. It is because the crack initiation life of specimens with deeper pits contributed to a smaller proportion of the total fatigue life. At this point, the impact of multiple fatigue sources is not so significant on the total fatigue life. In summary, the existence of corrosion and the coexistence of multiple pits on the fatigue life of steel wires should be considered.

#### 3.2.3. Fracture Toughness

Fracture toughness, a constant value, is an inherent property of a material that represents its resistance to crack propagation [39]. In this study, the maximum crack depth (*a_c_*) is applied to calculate the plane strain fracture toughness of the steel wire. As illustrated in Figure 13, starting from the center point of the circle formed by three points, the bisector of the crack propagation zone is chosen as the critical crack depth. The average diameter (*D_corr_*) and critical crack size (*a_c_*) at different corrosion levels on fatigue fracture surfaces are measured for the determination of the corrosion effect on the plane strain fracture toughness of the corroded steel wire.

**Figure 15:**
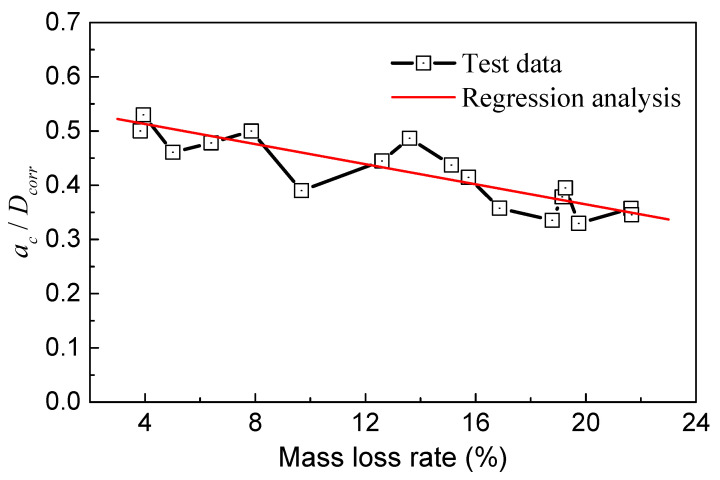
Relationship of *a_c_*/*D_corr_* and mass loss rate.

As illustrated in Figure 15, the ratio of *a_c_*/*D_corr_* decreased gradually with the mass loss rate. These two parameters roughly followed a linear relationship, indicating that the critical point of the crack propagation zone in the steel wire can move outward with increasing corrosion. As the mass loss became more severe, the proportion of the crack propagation zone area in the steel wire decreased. When the mass loss rate was around 4%, *a_c_*/*D_corr_* ranged from 0.5 to 0.55, and the change was not significant. *a_c_*/*D_corr_* began to decrease at a mass loss rate of 10%. When the mass loss rate reached 20%, *a_c_*/*D_corr_* decreased to a range of 0.3 to 0.4.

The reduction in *a_c_*/*D_corr_* alone cannot reflect the decrease in plane strain fracture toughness because the cross-sectional loss leads to an increase in tensile stress with the level of corrosion. During the fatigue cycling process, the cyclic stress can accumulate damage to the material and fractures in the steel wire will occur when the stress intensity factor (*K_IC_*) at the crack tip exceeds the critical value [40]. Equation (9) is adopted for the calculation of the fracture toughness of the corroded steel wire:

(9) $$K_{IC}=Y\left(\frac{a_{c}}{D_{corr}}\right)\sigma_{\max}\sqrt{\pi a_{c}}$$

where *Y* is the shape factor that is calculated by Equation (10) [41].

(10) $$Y\left(\frac{a_{c}}{D_{corr}}\right)=0.92\frac{2}{\pi }\sqrt{\frac{2D}{\pi a_{c}}\tan\frac{2D}{\pi a_{c}}}\frac{0.752+1.286\frac{a_{c}}{D_{corr}}+0.37\left(1-\sin\frac{\pi a_{c}}{2D_{corr}}\right)}{\cos\frac{\pi a_{c}}{2D_{corr}}}$$

**Figure 16:**
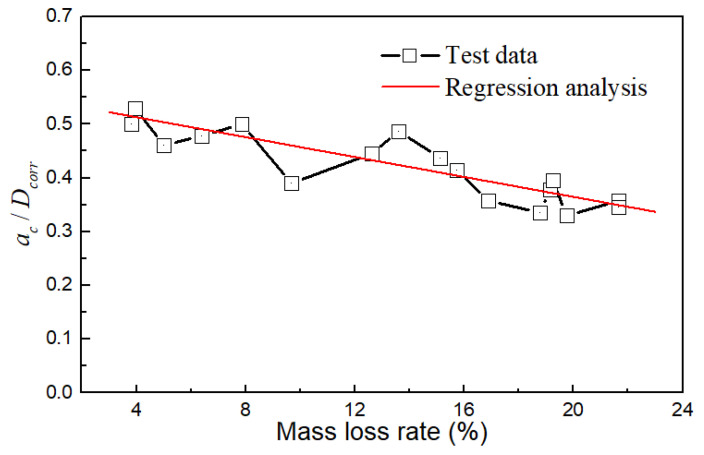
Influence of corrosion degree on fracture toughness.

Figure 16 depicts the plane strain fracture toughness at different mass loss rates. It is observed that the plane strain fracture toughness ($K_{IC}$) of the corroded steel wire had a negative correlation with the mass loss rate (*r*), following a linear pattern [42]. The determination coefficient (R^2^) was approximately 0.824, indicating a strong correlation between the two variables. The data points were well scattered within the 95% confidence interval band. The fitted equation relating fracture toughness and the mass loss rate is as follows:

(11) $$K_{IC}=104.5-2.132r$$

Taking the trend line of plane strain fracture toughness as an example, the corrosion effect in the absence of stress had a reducing impact on the fracture toughness of the steel wire. Note that this effect was insignificant in the early stage. The fracture toughness data fluctuated around 90 MPa·m^0.5^ before the mass loss rate reached 8%. After this point, the fracture toughness began to decrease. When it reached 20%, the fracture toughness data fluctuated around 60 MPa·m^0.5^. The main reason for the decrease was the electrochemical reactions during the early stages of salt spray corrosion test. A higher mass loss rate indicated longer exposure to corrosion. Electrochemical hydrogen evolution reactions in acidic environments and hydrogen permeation resulting from chemical reactions contributed to hydrogen diffusion inside the steel wire. Those reactions led to material embrittlement. The severity of embrittlement increased with a higher corrosion degree. Therefore, the decrease in fracture toughness under high corrosion rates should be considered in the evaluation of the service life of steel wires used by bridges in a corrosive environment.

## 4. Conclusions

This paper investigates the fatigue and mechanical behaviors of corroded bridge wires with Zn-Al alloy coating based on salt spray corrosion and fatigue tests. Based on the salt spray corrosion test and microstructure analysis, the anti-corrosion resistance and corrosion appearance characteristics of the Zn-Al alloy coating and galvanized coating were investigated. The fatigue and mechanical properties of the bare wires after the salt spray test were further studied based on tensile tests and fatigue tests. Fracture surfaces of the wires were captured and analyzed based on a method for recognizing graphical contours. The degradation of mechanical and fatigue properties subjected to different corrosion degrees, as well as fatigue life, was analyzed. The following conclusions can be drawn from the present testing and analysis.

(1) The superiority of the Zn-Al alloy coating in resistance to corrosion fatigue was confirmed by the analysis of the microstructure and salt spray corrosion test. The element Al can improve toughness and corrosion resistance for the generation of denser Zn-Al and Fe-Zn-Al phases. The salt spray resistance of the Zn-Al alloy coating was about twice more than that of the galvanized coating with the same thickness. A two-stage model was developed to evaluate the corrosion ability of the Zn-Al alloy coating, which can reach 29.1 years with a thickness of 70 μm.
(2) The mechanical properties of the bare wires after the salt spray test were further studied based on tensile tests, where the mass loss rates ranged from 0 to 21.38%. The test results show that the Young’s modulus, elongation rate, ultimate strain, tensile strength, and yield strength of the wire decreased as the mass loss rate increased and the most obvious change was in elongation rate. The corrosion effect was mainly manifested in the ductility of the Zn-Al alloy-coated wires, and the ultimate strain gradually decreased with the corrosion degree.
(3) The corrosion fatigue fracture surfaces were analyzed using a high-precision industrial camera and by recognizing graphical contours. Fatigue initiation sites were mostly located near the bottom of the pits rather than at the interface between the pits and the steel wire surface. Insufficient fatigue life may occur in the steel wires used in actual engineering after corrosion for the interaction of fatigue stress and corrosion action. This impact increased with the degree of corrosion. Corrosion fatigue was more sensitive to the existence of fatigue sources than the reduction in the cross-section caused by corrosion. The pit depth logarithmically weakened the fatigue life of steel wires for weakening the fatigue toughness and bearing area. The presence of multiple initiation sources significantly reduced the fatigue life due to the cracking facilitation of the joint effect of multiple pits. The flat fracture was more common with a single fatigue source, while multiple fatigue sources led to step-like fractures for the generation of multiple dispersed crack propagation regions.
(4) Corrosion had a reducing effect on the fracture toughness of the steel wires, which can be described adequately by a linear model. The fracture toughness of the corroded Zn-Al alloy-coated wire was calculated to decrease from 90 MPa·m^0.5^ at the mass loss rate of 8% to 60 MPa·m^0.5^ at the mass loss rate of 20%. This is probably because the electrochemical reactions of corrosion can lead to material embrittlement and a decrease in fracture toughness, which should be of concern in the fatigue life evaluation.

## Figures and Tables

**Table 1:** Chemical composition of the bare steel wires studied here.

Composition	Iron (Fe)	Carbon (C)	Silicon (Si)	Manganese (Mn)	Chromium (Gr)	Cuprum (Cu)	Sulfur (S)
Weight percent (%)	>98.0	0.8–0.85	0.15–1.00	0.60–0.90	≤0.20	≤0.06	≤0.025

**Table 2:** Mechanical properties of steel wires with galvanized coating and Zn-Al alloy coating.

Wire Type	Tensile Strength (MPa)	Yield Strength (MPa)	Elongation Rate (%)	Elastic Modulus (GPa)
Galvanized	1835 ± 67	1620 ± 64	6.8 ± 0.5	203 ± 23
Zn-Al coated	1935 ± 71	1706 ± 67	6.3 ± 0.5	204 ± 23

## Data Availability

The data presented in this study are available on request from the corresponding author. The data are not publicly available due to privacy or ethical reasons.
